# Peritoneal cell-free DNA as a sensitive biomarker for detection of peritoneal metastasis in colorectal cancer: a prospective diagnostic study

**DOI:** 10.1186/s13148-023-01479-9

**Published:** 2023-04-18

**Authors:** Zixu Yuan, Wenle Chen, Duo Liu, Qiyuan Qin, William M. Grady, Alessandro Fichera, Huaiming Wang, Ting Hou, Xinze Lv, Chanhe Li, Hui Wang, Jian Cai

**Affiliations:** 1 Present Address: Department of Colorectal Surgery, Department of General Surgery, Guangdong Institute of Gastroenterology, Guangdong Provincial Key Laboratory of Colorectal and Pelvic Floor Diseases, The Sixth Affiliated Hospital, Sun Yat-Sen University, No. 26 Yuancun Erheng Rt, Tianhe District, Guangzhou, 510655 China; 2 Department of Colorectal Surgery, Zhongshan Municipal People’s Hospital, Zhongshan, China; 3 Clinical Research Division, Fred Hutchinson Cancer Research Center, Seattle, WA USA; 4 Colon and Rectal Surgery, Baylor University Medical Center, Dallas, TX USA; 5 Burning Rock Biotech, Guangzhou, China; 6 Department of Colorectal Surgery, The First Affiliated Hospital of Shenzhen University, Shenzhen Second People’s Hospital, No. 3002 Sungang West Road, Futian District, Shenzhen, Guangdong Province China

**Keywords:** Peritoneal metastasis, Colorectal cancer, Cell-free DNA, NGS, Driver mutations

## Abstract

**Background:**

The detection of peritoneal metastasis (PM) is limited by current imaging tools. In this prospective study, we aimed to evaluate the sensitivity and specificity of peritoneal cell-free DNA (cfDNA) for diagnosis of PM.

**Methods:**

Colorectal cancer (CRC) patients with/without PM were enrolled. The cfDNA experimental personnel and statists were blinded to the diagnosis of PM. Ultradeep sequencing covering large genomic regions (35000X, Next-generation sequencing) of cfDNA in peritoneal lavage fluid (FLD) and matched tumor tissues was performed.

**Results:**

A total of 64 cases were recruited prospectively and 51 were enrolled into final analysis. In training cohort, 100% (17/17) PM patients obtained positive FLD cfDNA, comparing to 5/23 (21.7%) in patients without PM. Peritoneal cfDNA had a high sensitivity of 100% and specificity of 77.3% for diagnosis of PM (AUC: 0.95). In validation group of 11, 5/6 (83%) patients with PM obtained positive FLD cfDNA, comparing to 0/5 in non-PM (*P* = 0.031) with a sensitivity of 83.3% and specificity of 100%. Positive FLD cfDNA was associated with poor recurrence-free survival (*P* = 0.013) and was preceding radiographic evidence of recurrence.

**Conclusions:**

Peritoneal cfDNA is a promising sensitive biomarker for earlier detection of PM in CRC than current radiological tools. It can potentially guide selection for targeted therapies and serve as a surrogate instead of laparoscopic explore in the future.

*Trial Registration* Chinese Clinical Trial Registry at chictr.org.cn (ChiCTR2000035400). URL: http://www.chictr.org.cn/showproj.aspx?proj=57626

**Supplementary Information:**

The online version contains supplementary material available at 10.1186/s13148-023-01479-9.

## Background

Colorectal cancer (CRC) is one of the most common cancers worldwide, ranking the third position of new cases and deaths of all cancers [1]. Peritoneal metastasis (PM) is considered to be the terminal stage of CRC (stage IVc) due to poor prognosis according to TNM staging system. The incidence of synchronous PM is 17–40%, while the incidence of metachronous PM is 44–50% [2]. Unfortunately, conventional clinical assessment and current imaging tools are not sufficiently robust to detect PM. The sensitivity of CT in diagnosis of PM for small amounts of metastatic disease (diameter < 5 mm implants) is only 11%, and similar diagnostic power using PETCT [3, 4]. PM can be treated by cytoreductive surgery (CRS) and hyperthermic intraperitoneal chemotherapy (HIPEC). Early PM of CRC can achieve good prognosis after CRS plus HIPEC with a median OS of 22–40 months [5]. However, patients with extensive PM (or late PM) with a peritoneal cancer index (PCI) > 20 are not appropriate for CRS, as no survival benefit of CRS and higher surgical complications of major surgery [6]. Recently, we have developed an image-based deep learning algorithm ResNet 3D by artificial intelligence for detection of PM [3]. Accordingly, there is an urgent clinical need to develop more sensitive tools for early diagnosis of PM.

Preliminary study has suggested the feasibility of cfDNA detection in peritoneal ascites and peritoneal lavage fluid (FLD) [7]. However, no study to date has adopted ultradeep next-generation sequencing (NGS) to detect peritoneal cfDNA and investigate the diagnostic power of cfDNA for PM. Here, we report the results of the sensitivity and specificity of peritoneal cfDNA in diagnosis of PM. Peritoneal cfDNA can be used as a promising biomarker in postoperative surveillance and be adjuvant tool for diagnostic laparoscopy to detect peritoneal disseminations in high-risk CRC.

## Results

### Clinicopathological characteristics

**Fig. 1:**
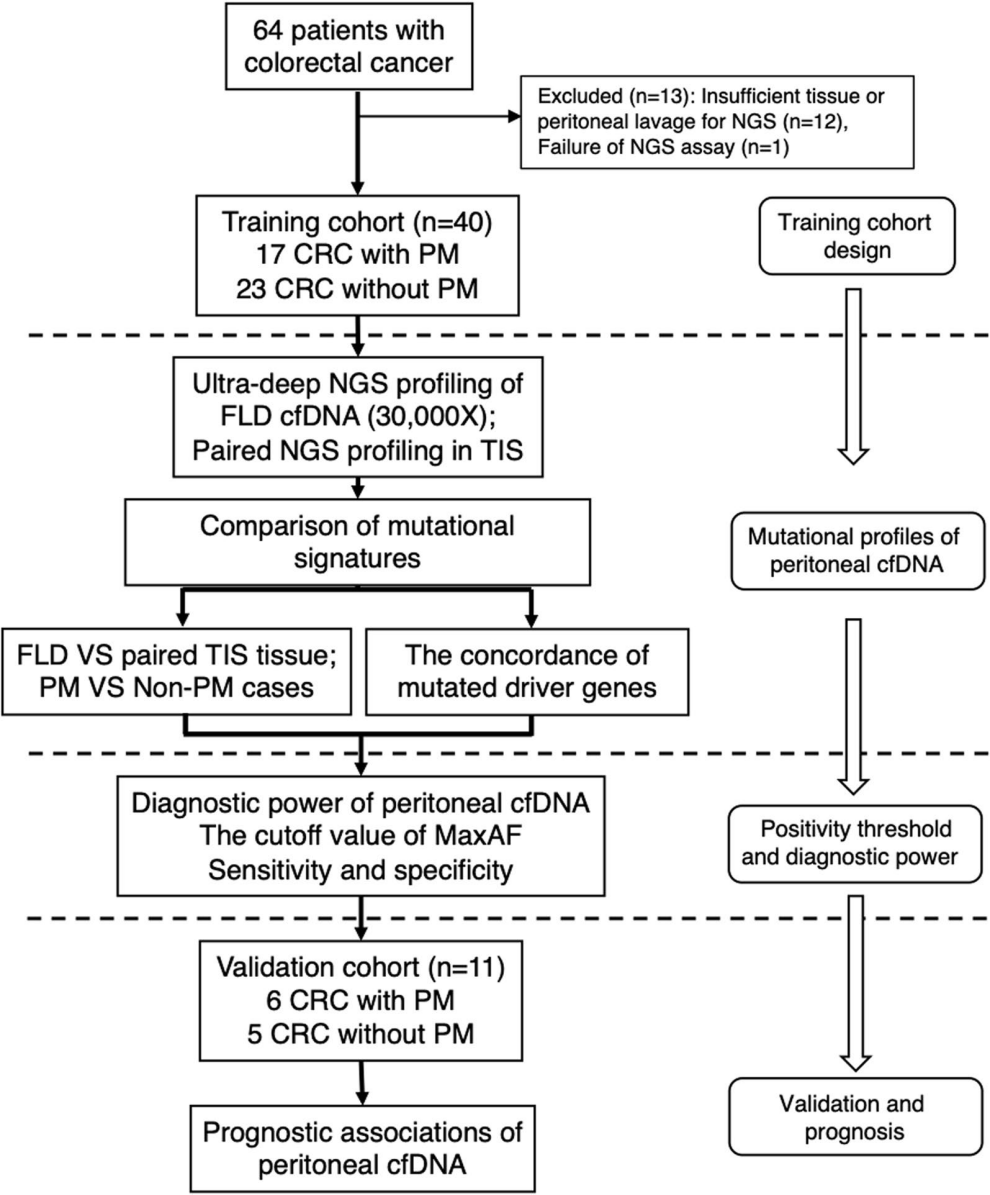
The workflow of patient enrollment in this study. 40 CRC patients were including in the training cohort underwent NGS assays for mutational profiles of peritoneal cfDNA and 11 CRC patients were enrolled in the validation cohort

A total of 64 consecutive patients with CRC were recruited prospectively. Thirteen cases were excluded for subsequent analysis, due to insufficient tissue or peritoneal lavage for NGS assays (*n* = 12), and failure of NGS assay (*n* = 1). 51 cases were finally enrolled into primary analysis in this study. NGS profiling of cfDNA in FLD and DNA mutations in tumor tissue (TIS) were available in 40 cases in the training cohort, which consisted of 17 PM and 23 non-PM cases. The remaining 11 cases were enrolled in the validation cohort and NGS were conducted only in FLD as part of studies to validate the positivity threshold of MaxAF value in discrimination of PM from non-PM (Fig. 1)*.* Patient’s demographics of overall 51 and 40 in the training cohort were summarized (Table 1 and Additional file 1: Table S1). The median age was 56 years old and 62% (25/40) were females. The mean PCI score in PM was 17 points (range: 9 ~ 22). Serum CA125 and CA199 were elevated in PM than non-PM (*P* < 0.05) (Table 1).

**Table 1:** The clinicopathological characteristics of enrolled patients in the overall and training cohort

	Overall (*n* = 40)	CRC with PM (*n* = 17)	CRC without PM (*n* = 23)	*P* value
*Gender*				
Female	25 (62.5)	13 (76.5)	12 (52.2)	0.187
Male	15 (37.5)	4 (23.5)	11 (47.8)	
*Age*				
Mean(SD)	53.73 (10.50)	51.53 (11.81)	55.35 (9.35)	0.33
*Histological type*				
Adenocarcinoma	35 (87.5)	16 (94.1)	19 (82.6)	0.624
Mucinous adenocarcinoma	3 (7.5)	1 (5.9)	2 (8.7)	
Signet ring cell carcinoma	2 (5.0)	0 (0.0)	2 (8.7)	
*Location*				
Left	28 (70.0)	9 (52.9)	19 (82.6)	0.079
Right	12 (30.0)	8 (47.1)	4 (17.4)	
*Alcohol tobacco*				
No	36 (90.0)	14 (82.4)	22 (95.7)	0.294
Yes	4 (10.0)	3 (17.6)	1 (4.3)	
*TNM_T*				
T1	2 (5.0)	0 (0.0)	2 (8.7)	< 0.001
T2	1 (2.5)	0 (0.0)	1 (4.3)	
T3	23 (57.5)	5 (29.4)	18 (78.3)	
T4	10 (25.0)	9 (52.9)	1 (4.3)	
Tis	1 (2.5)	0 (0.0)	1 (4.3)	
Tx	3 (7.5)	3 (17.6)	0 (0.0)	
*TNM_N*				
N0	20 (50.0)	6 (35.3)	14 (60.9)	0.154
N1	10 (25.0)	5 (29.4)	5 (21.7)	
N2	7 (17.5)	3 (17.6)	4 (17.4)	
Nx	3 (7.5)	3 (17.6)	0 (0.0)	
*TNM_M*				
M0	18 (45.0)	0 (0.0)	18 (78.3)	< 0.001
M1	22 (55.0)	17 (100.0)	5 (21.7)	
*MSI status*				
MSI-H	1 (2.5)	0 (0.0)	1 (4.3)	1.0
MSS	39 (97.5)	17 (100.0)	22 (95.7)	
*TMB in TIS*				
Mean(SD)	5.43 (7.13)	3.93 (1.75)	6.55 (9.21)	0.294
*PCI scores*				
Mean(SD)	7.40 (11.38)	17.41 (11.42)	0.00 (0.00)	< 0.001
*CA125 (ng/ml)*				
Median[IQR]	19.40 (10.47,34.17)	35.00 (10.50, 102.20)	14.50 (10.95, 20.55)	0.027
*CA199 (ng/ml)*				0.016
Median[IQR]	16.00[7.02, 47.60]	45.17[10.26, 208.21]	9.89[4.32, 26.75]	
*CEA (ng/ml)*				0.103
Median[IQR]	7.91[3.07, 22.14]	10.84[4.14, 43.00]	6.11[2.88, 12.31]	

### The concordance of gene mutations in peritoneal lavages and tumor tissues

**Fig. 2:**
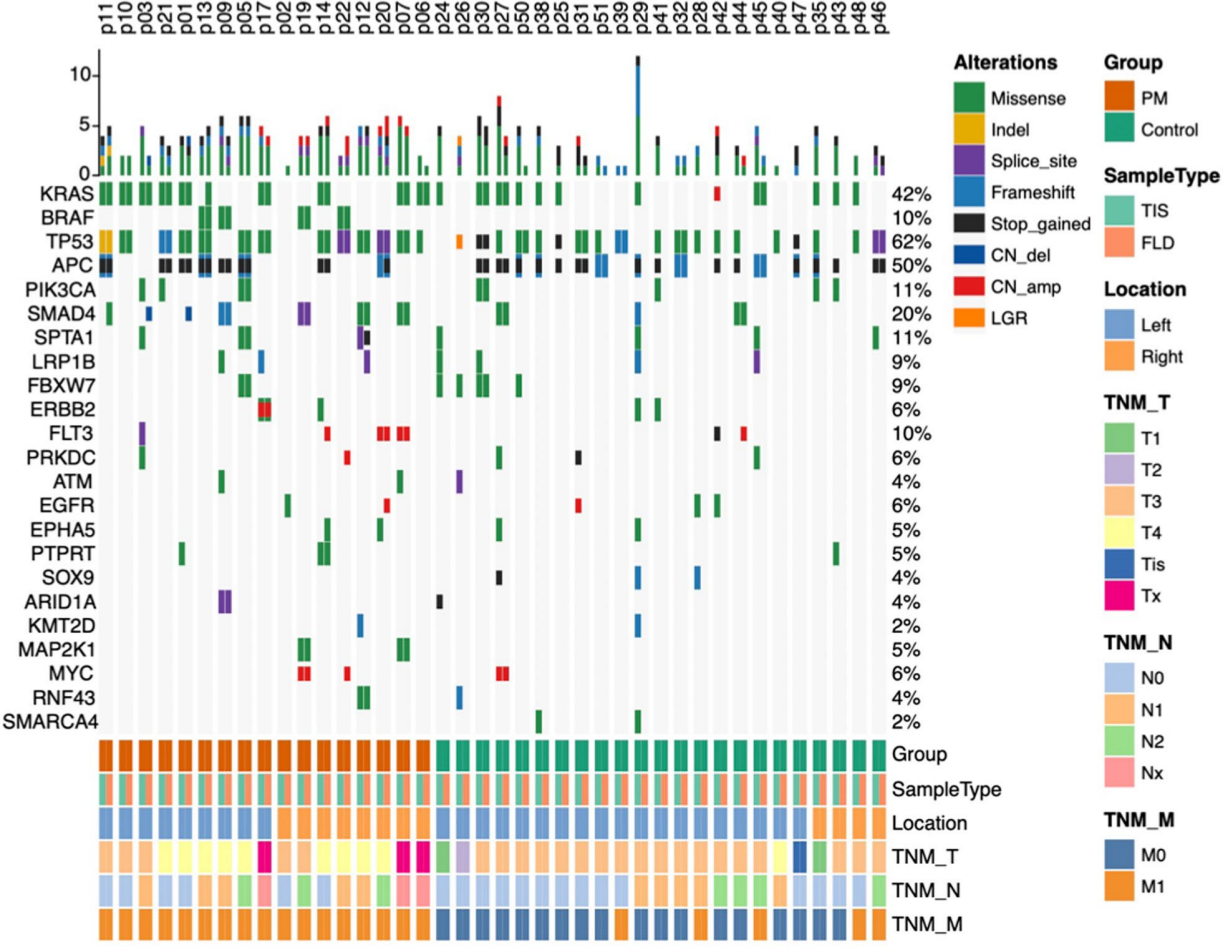
The mutational profiling of cfDNA in matched FLD and TIS of PM and non-PM patients were compared by cluster plot. More mutations were shown in both FLD and TIS of PM patients, while no mutant in FLD in the major of non-PM patients were observed. The top five frequent gene mutations were KRAS (44%), TP53 (62%), APC(50%), PIK3CA(11%) and SMAD4(20%). Alleviations: TNM-TNM staging system, CN- copy number, TIS- tissue sample, FLD- peritoneal lavage fluid

**Fig. 3:**
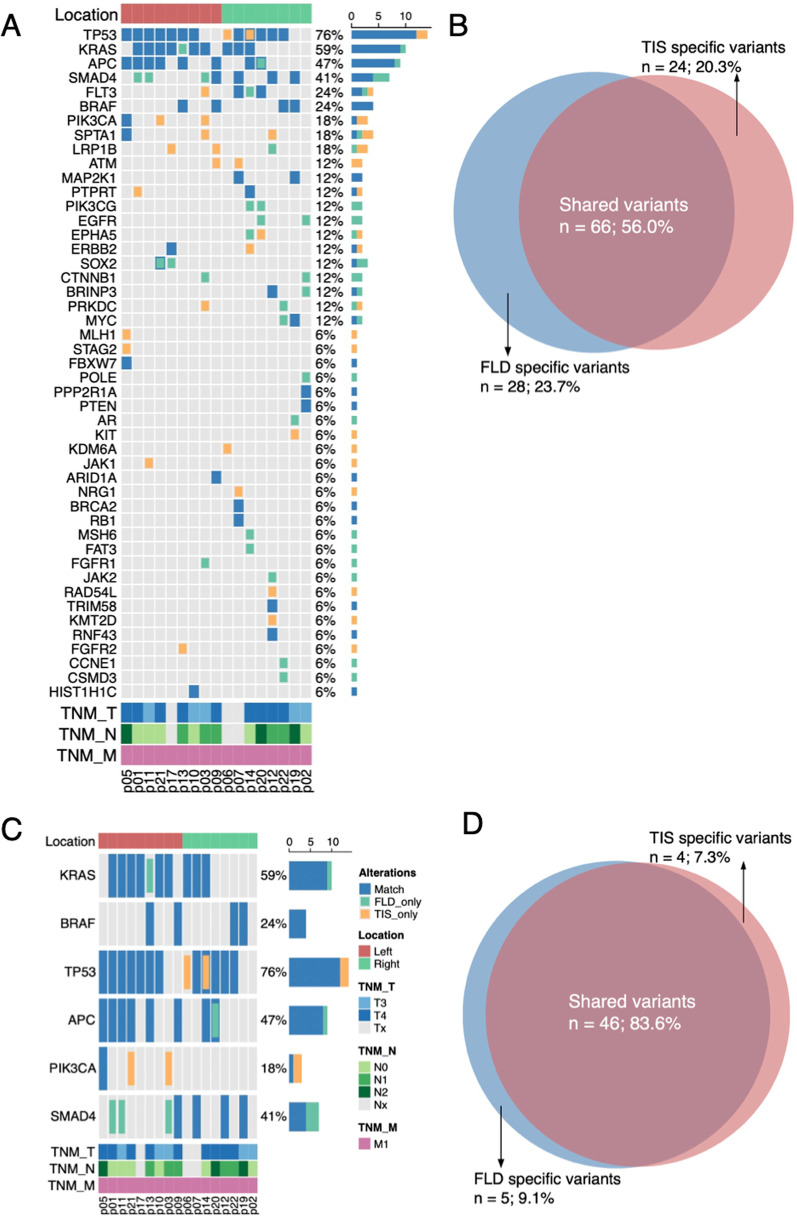
The high concordance of gene mutations between peritoneal lavages and tumor tissues in PM, especially driver genes. **A**, **B** 56.0% (*n* = 66) of shared variant in PM were observed both in FLD and TIS. **C**, **D** 83.6% (*n* = 46) of driver gene mutations including KRAS/BRAF/TP53/APC/PIK3CA/SMAD4 in PM were shared both in FLD and TIS with 92% sensitivity and 100% specificity

The sequencing depth and DNA insert sizes fulfilled the quality of control in both FLD and TIS of PM and non-PM (Additional file 2: Fig. S1). In the training cohort, genetic mutational profiles in FLD and TIS were compared. The frequencies of overall mutation were lower in FLD than TIS due to few mutations being detected in FLD of non-PM patients (Fig. 2 and Additional file 2: Figs. S2, S3). As for specific genetic variants, in 12 out of 40 patients the same missense mutation of the KRAS gene was found both in TIS and FLD, in 10 out of 40 patients mutations were detected only in FLD or TIS, and 9 out of 10 were detected only in TIS. The proportion of consistent mutation sites in *BRAF, TP53, APC, PIK3CA, SMAD4* genes detected in TIS and FLD were 4/40, 18/40, 14/40, 2/40, 6/40, respectively, and other inconsistent mutations were more detected in TIS. Over a half of all altered variants (56.0%) including shared variants, FLD specific variants and TIS specific variants in PM were shared both in FLD and TIS, and other 20.3% of TIS specific variants and 23.7% of FLD specific variants were observed with a clinical sensitivity of 73% (Fig. 3A, B). The majority of altered variants (83.6%) of driver genes in PM were shared both in FLD and TIS, and other remaining 7.3% of TIS specific variants and 9.1% of FLD specific variants (Fig. 3C, D) were found. In addition, 58.9% of shared single nucleotide variants (SNV)/Indel variants were shown both in FLD and TIS in PM with sensitivity of 72% (Additional file 2: Fig. S4). As expected, more mutations were shown in both FLD and TIS of PM patients, while no mutations in FLD were observed in the majority of non-PM patients (Fig. 2). Thus, high concordance of gene mutations between FLD and TIS were shown and detection of FLD cfDNA could reflect tumor mutant features.

### The mutational profiling of cfDNA of driver gene in peritoneal and tumor tissues

**Fig. 4:**
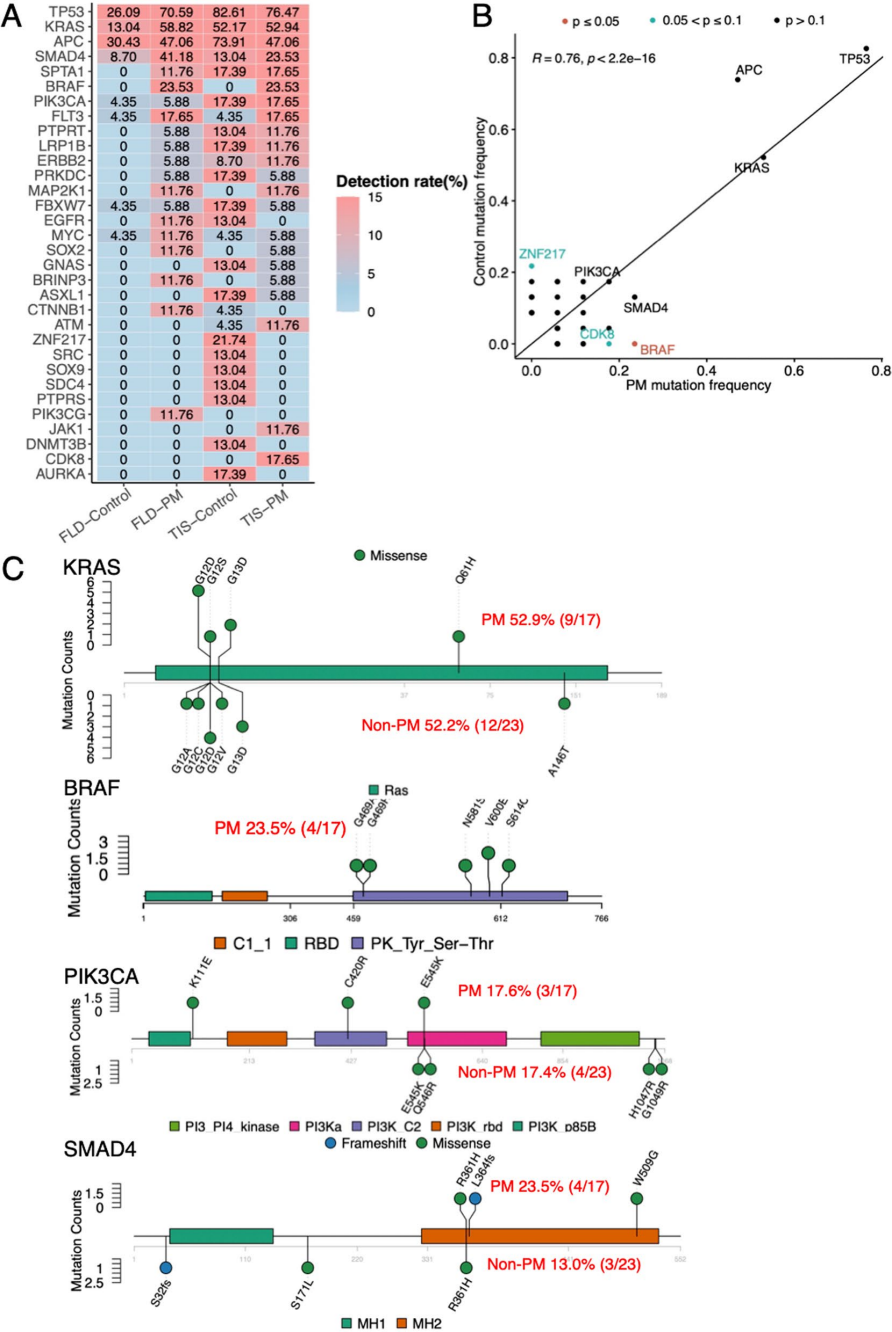
The mutational frequencies and sites of driver genes in peritoneal cfDNA and tumor tissues of both PM and non-PM. **A** The detected frequencies of driver gene mutations were consistent in FLD (FLD-PM) and TIS (TIS-PM) of PM, especially TP53, KRAS, APC and BRAF. **B** In tumor tissue, BRAF mutations were only observed in PM (*P* < 0.05). **C** In tumor tissues, 52.9% (9/17) of KRAS mutations, 23.5% (4/17) of BRAF mutations, 17.6% (3/17) of PIK3CA mutation and 23.5%(4/17) of SMAD4 mutation were all observed in PM. Only BRAF mutations were significantly higher in PM than non-PM (*P* < 0.05)

We investigated the mutational frequencies of driver genes in both TIS and FLD of PM and non-PM. The top driver mutations in FLD-PM were suppressor gene *TP53* (70%), *APC* (47%) and oncogenic *KRAS* (58%) and *SMAD4* (41%) (Fig. 4A). The frequencies of driver mutations in FLD cfDNA were consistent with TIS cfDNA in the PM. However, lower frequencies of driver mutations or no mutations were observed in FLD comparing to TIS in non-PM due to low mutational burden of FLD in cases without PM (Fig. 4A). Thus, FLD cfDNA was a sensitive and accurate biomarker for detection of driver mutations in PM, similar to TIS, but not for non-PM.

In scatter plots of driver gene concordance in tissues, *BRAF* mutations were only observed in PM, which indicated the close relationship between *BRAF* mutations and PM (*P* < 0.05) (Fig. 4A, B). 17.7% of *CDK8* mutations were observed in TIS-PM comparing to 0 in TIS-control (*P* = 0.06). 21.7% of *ZNF217* mutations were found in TIS-control comparing to 0 in TIS-PM (*P* = 0.06) (Fig. 4A, B). In particular, oncogenic *KRAS* mutations were detected in 52.9% (9/17) in TIS of PM, comparing to 52.2% (12/23) in non-PM. 23.5% (4/17) of oncogenic BRAF mutations were identified in TIS of PM, including V600E and G469X mutations. Mutant *PIK3CA* was detected in 17.6% (3/17) of PM, comparing to 17.4% (4/23) of non-PM. 23.5% (4/17) of *SMAD4* mutations (*WNT* Signaling) were observed in TIS of PM, comparing to those identified in 13.0% (3/23) in TIS of non-PM (Fig. 4C). The mutational frequencies and types of suppressor *TP53* and *APC* were listed in Additional file 2: Fig. 5. In the previous study, amplification in *Chr20q* (Microsatellite Stable (*MSS)*-A) was the most common copy number variant *(CNV)* and predicted better prognosis in CRC [8]. In this study, 100% (17/17) of PM were *MSS-N,* comparing to 81.8% (18/22) of non-PM with a non-significant trend (*P* = 0.118) (Additional file 2: Fig. S5). These results suggested different frequencies of driver mutations in tumor tissues of PM and non-PM.

Furthermore, we investigated whether the Maximum allele frequency (MaxAF) values could be affected by single driver mutation in FLD of PM and non-PM. No significant differences of average MaxAF were shown (*P* > 0.05) in wild-type and mutant driver genes in the FLD of PM, including K*RAS, BRAF, TP53, APC, PIK3CA, and SMAD4* (Additional file 2: Fig. S6). In the FLD of non-PM, higher MaxAF were observed in mutant *TP53* (*P* = 0.02), mutant *APC* (*P* < 0.01) and *SMAD4* (*P* = 0.05) than wild types (Additional file 2: Fig. S6). These data showed single driver mutation could not affect the FLD MaxAF due to overall high frequencies of mutations in PM, while some driver mutations could increase the MaxAF due to overall low frequencies of mutations in cases without PM.

### Altered signaling pathways and key genes in PM

To clarify the underlying mechanism of PM, we conducted KEGG enrichment analysis to identify genetic mutational variations in the tumor tissues of PM and non-PM. The top signaling of mutant genes were comparable in PM and non-PM, including *MAPK, WNT, and ERBB* signaling (Additional file 2: Fig. S7). The details of KEGG enrichment analysis was listed in Additional file 1: Table S3. The top five genes in these signaling were T*P53, APC, EGFR, KRAS, and PIK3CA*.

### The cutoff value and diagnostic power of positive FLD cfDNA

**Fig. 5:**
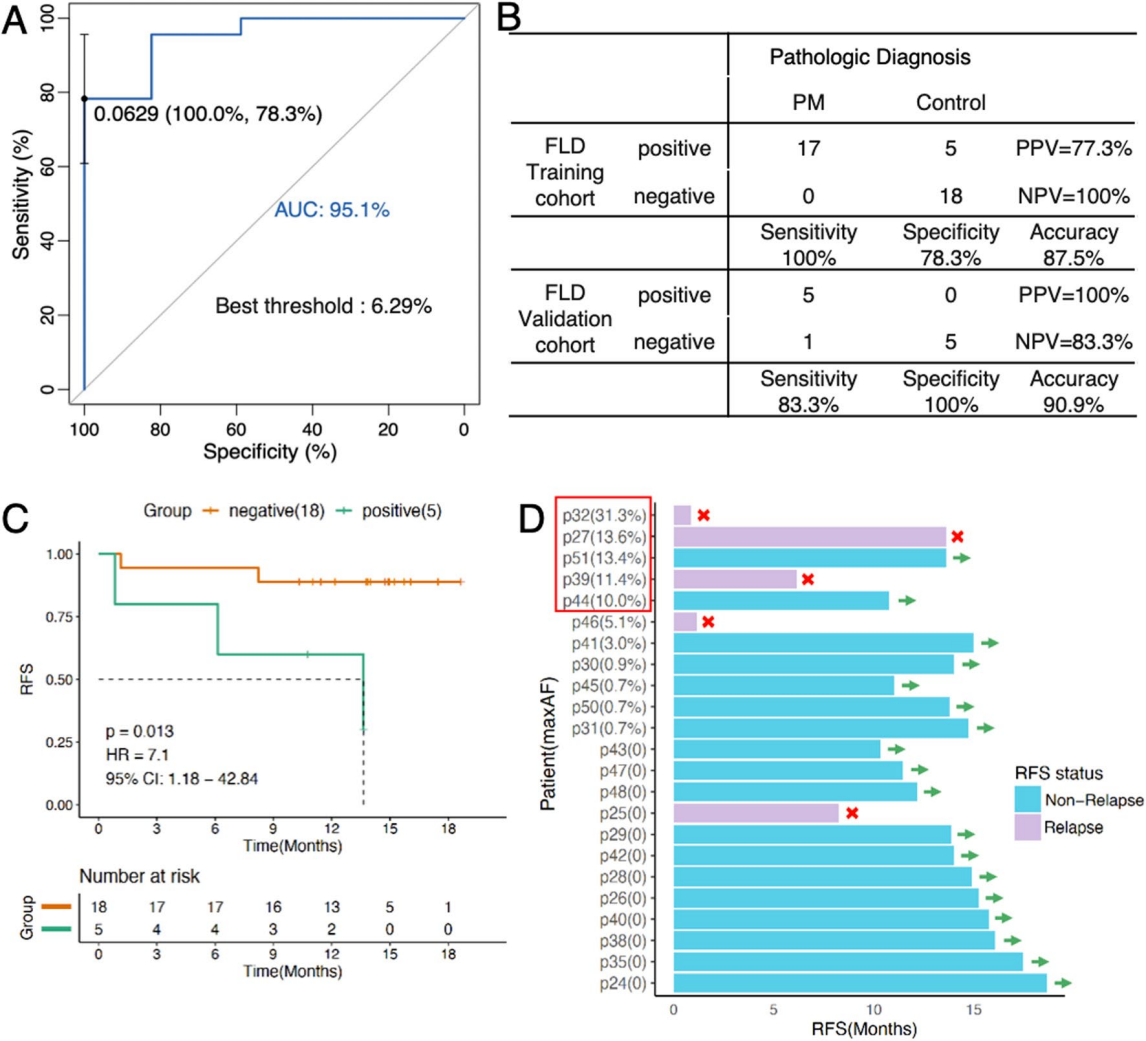
The accuracy of peritoneal cfDNA for detection of PM and prediction of recurrence. **A** The cutoff value of MaxAF was identified at best threshold of 6.29% (AUC: 0.951) with 100% of sensitivity and 78.3% of specificity by ROC analyses. **B** In the training cohort, the sensitivity, specificity and accuracy of peritoneal cfDNA for PM detection were 100%, 78.3%, 87.5%, respectively. In the validation cohort, 83.3% of sensitivity, 100% of specificity and 90.9% of accuracy were shown. **C** Positive peritoneal cfDNA was associated with tumor recurrence (HR = 7.1 (95% CI: 1.18, 42.84), *P* = 0.013). **D** During follow-up, recurrence occurred in 3/5 (60%) of CRC patients with positive cfDNA (mean MaxAF 18.7%), while only 2/18 (11.1%) recurrences of lung metastasis were observed in CRC patients with negative cfDNA (*P* = 0.083)

To assess the clinical utility of peritoneal cfDNA in diagnosis of PM, we identified the cutoff value of FLD MaxAF at best positivity threshold of 6.29% performed by ROC analyses with area under curve (AUC) of 0.951 in the training cohort (Fig. 5A). In addition, we found the distribution of blood MaxAF, in a large Chinese cohort of CRC from the bio-database of Burning Rockstone company, were lower than this positive cutoff value, which can filter the majority of germline variants (Additional file 2: Fig. S8). Over the positivity threshold was classified as positive and otherwise below was negative. In the training cohort, all 17 patients with PM (100%) obtained positive FLD cfDNA, comparing to 5/23 (21.7%) in patients without PM. The sensitivity, specificity and accuracy of peritoneal cfDNA in detection of PM were 100%, 78.3%, 87.5%, respectively. The PPV and NPV were 77.3% and 100%, respectively (Fig. 5B). In addition, recurrence occurs in 3/5 (60%) of non-PM cases with positive peritoneal cfDNA (Fig. 5D), namely false-positive or micrometastatic disease (MRD) during follow-up.

To verify the diagnostic power and accuracy of peritoneal cfDNA in detection of PM, we conducted NGS assays in the FLD of randomized CRC patients in a validation cohort of 11 patients. In the validation cohort, 5/6 (83%) patients with PM obtained positive FLD cfDNA, comparing to 0/5 in patients without PM (*P* = 0.031) (Fig. 5B). Peritoneal cfDNA in 5 of PM cases displayed MaxAF value over the positivity threshold (mean MaxAF > 40%), while only one of PM patients (MaxAF 0) was below the threshold who was affected by neoadjuvant chemotherapies. In addition, 5/5 (100%) of non-PM patients were presented with negative peritoneal cfDNA below the threshold (all MaxAF was 0) (Additional file 2: Fig. S9). The clinical sensitivity, specificity, accuracy of peritoneal cfDNA in detection of PM were 83.3%, 100%, and 90.9%, respectively (Fig. 5B). Together, these above data showed high performance of peritoneal cfDNA in diagnosis of PM.

We investigated the associations between peritoneal cfDNA and clinicopathological features in PM. No significant associations were observed between FLD MaxAF and these clinicopathological parameters including age, gender, alcohol & tobacco, tumor location, CEA, CA199, CA125, PCI scores, T stage, N stage, and histological type. However, CA153 levels was positively correlated with FLD MaxAF (*P* < 0.05) (Additional file 1: Table S2).

### Peritoneal cfDNA and RFS

To clarify the prognostic value of peritoneal cfDNA in CRC patients without PM, 23 CRC patients in the non-PM were followed-up from the date of surgery until tumor recurrence or until Aug 31, 2022 for patients without tumor relapse. The FLD cfDNA was sampled at the aspiration of peritoneal fluid during the surgery. The median follow-up time was 13.8 months (range: 0.8–18.6 months). Positive peritoneal cfDNA was associated with tumor recurrence (*P* = 0.013, HR: 7.1, 95% CI: 1.18–42.84) (Fig. 5c), especially in stage III of CRC (*P* = 0.043) (Additional file 2: Fig. S9). Univariant and multivariant analyses also revealed significant associations between positive cfDNA and shorter RFS (Additional file 1: Table S2). 5 cases (3 with positive peritoneal cfDNA and two with negative cfDNA) experienced tumor relapses confirmed by imaging findings. The median time to relapse from surgery was 7.1 months (Fig. 5D). Among them, recurrence occurred in 3/5 (60%) with positive peritoneal cfDNA (mean MaxAF 18.7%, range: 31.3, 13.6 and 11.4%), including one patient of PM and two patients of hepatic metastasis by contrast CT (PM may exist due to lack of laparoscopic exploration during follow-up). Detection of cfDNA preceded radiological findings by contrast CT with a lead time of 409, 184, and 25 days (median: 206d), respectively. The other two recurrences of both lung metastasis (2/18 (11.1%), MaxAF 5.1% and 0), were observed among 18 cases with negative peritoneal cfDNA (mean MaxAF 0.6%) (Fig. 5D). These results revealed higher MaxAF values of peritoneal cfDNA presented more probabilities of recurrence and peritoneal cfDNA was a promising accurate and earlier prognostic biomarker in surveillance of CRC recurrence.

## Discussion

In this prospective study, we reported that peritoneal cfDNA in lavage fluid by ultradeep NGS was a reliable tool for detection of PM in CRC with high sensitivity and specificity. High concordance of genetic mutational profiling and driver genes were observed between peritoneal cfDNA and tumor tissue of CRC patients. The clinical utility of peritoneal cfDNA was proven in the validation cohort. Therefore, the importance of peritoneal cfDNA as a liquid biopsy proves to be a surrogate or adjuvant biomarker instead of laparoscopy for earlier diagnosis of PM.

Early detection of PM in CRC is a difficult clinical issue. CEA is current tumor biomarker in detection of tumor recurrence, but is limited by low sensitivity and specificity [9]. Current imaging tools are limited to detect small peritoneal metastatic nodules with diameter < 5 mm with 11–48% of sensitivity by enhanced CT [4]. MRI with DWI sequences or PETCT can improve the performance but not enough to detect occult micro metastatic nodules [4]. Early diagnosis of PM with PCI < 20 can be treated with CRS plus HIPEC with an OS of 42 months according to PRODIGE 7 trial [5]. However, patients with extensive PM obtained poor prognosis with an OS less than one year. Recently, we have developed an image-based deep learning algorithm by artificial intelligence for detection of synchronous PM with 94% of accuracy [3].

Currently, interest has developed in the usage of liquid biopsy for cfDNA in early detection of cancers [10]. cfDNA carrying tumor-derived sequence alterations apart from germline mutations that attributes to clonal hematopoiesis are identified in the blood [11], peritoneal ascites [12], and cerebrospinal fluid [13]. Surveillance of tumor recurrence and genotyping using cfDNA offers potential advantages especially in metastatic disease [14]. In prior studies, cfDNA serves as one more sensitive biomarker than CEA for recurrence surveillance and is an earlier biomarker than CT findings for recurrence detection in CRC [15, 16].

Primary tumor shed cfDNA into the circulation, and the concentration of plasma cfDNA is relatively high for metastatic or recurrent CRC [17]. Plasma cfDNA is not a sensitive biomarker to detect PM of CRC for lower levels of cfDNA than distant metastasis. This phenomenon was due to peritoneal dissemination instead of circulation and peritoneum-plasma barrier to restrict cfDNA release to circulation [18]. The origins of peritoneal cfDNA can better reflect tumor-derived cfDNA than plasma, due to less contamination of confound germline mutations in peritoneal fluid than plasma. Recently, Leick et al. [12] reported *KRAS* mutant DNA is detected in peritoneal fluid and can serve as potential biomarker of PM. In this study, peritoneal cfDNA is proven to be a reliable biomarker to detect PM and is clinically feasible. Laparoscopy is widely used and aspiration of ascites or peritoneal washing through drainage tube can obtain peritoneal cfDNA during follow-up in high-risk CRC.

To our knowledge, this is the first study to report peritoneal cfDNA for detection of CRC with PM. Peritoneal fluids are not common in CRC with early PM. Thus, we have conducted this cfDNA assays by peritoneal lavages. High similarity of gene mutations between peritoneal lavage and tumor tissue were shown in PM. Thus, mini-invasive peritoneal cfDNA could reflect tumor mutant burden and molecular features of PM instead of invasive test by tumor tissue. Ultradeep NGS can overcome the limitations of shallow sequencing depth of NGS profiling to achieve high sensitivity similar to ddPCR that is restricted to specific hotpot mutations [19]. The 100% of sensitivity in peritoneal cfDNA to detect PM highlight the advantages of diagnosis over current imaging tools and blood biomarkers CEA. In addition, recurrence occurs in 40% of non-PM cases with positive peritoneal cfDNA (false-positive or MRD) during follow-up. This suggests cfDNA can detect PM a few months preceding imaging findings for early detection. NGS-based cfDNA can serve as a potential surrogate or adjuvant biomarker for active surveillance of peritoneal dissemination instead of laparoscopic explore in high-risk CRC. During follow-up, physicians can insert drainage tubes into peritoneal cavity under ultrasound guide by local anesthesia. This peritoneal cfDNA assay can serve as adjunct tool to diagnostic laparoscopy for detection of microscopic residual disease.

Peritoneal FLD and matched TIS are set to decrease false-positive germline variants and identified tumor-derived mutations of cfDNA to filter out background signal that comes from non-tumor-derived mutations. Matched cfDNA-WBC sequencing is usually applied in plasma cfDNA studies to exclude non-tumor derived cfDNA from clonal hematopoiesis in the blood [20]. To exclude potential germline mutations, we also observed above 70% of MaxAF values in this study was below positivity threshold MaxAF 6.29% in more than 1000 plasma samples from the company database of CRC patients. The positivity threshold was efficient to detect PM and can reflect tumor-specific burden such as tumor metastasis or MRD instead of plasma cfDNA. The positivity threshold is high in this study (usually ~ 1% for NGS), because ultradeep NGS is too sensitive to detect mutations [20]. In addition, peritoneal cfDNA is negative in one patient of PM who underwent adjuvant chemotherapy. Theoretically, chemo reagents will eliminate cfDNA fragment both in the plasma and peritoneal fluids and can cause false-negative result [10, 19]. Tie et al. [15] reported circulating cfDNA of mCRC can be changed after chemotherapy.

High concordance of driver genes in peritoneal lavage and tumor tissues are identified in this study. *KRAS* mutant cfDNA is detected in peritoneal fluids similar to tumor tissue in previous study [12]. *BRAF* mutations was only found in PM in this study, which indicate the close correlation between *BRAF* mutation and PM. In clinical practice, the prognosis of patients with *BRAF* mutations are the poorest (especially *V600E* mutation), even worse than mutant *KRAS G12D/13D.* Recurrence and PM are commonly seen after radical surgery in CRC with BRAF mutant and chemotherapy are resistant in these patients. KEGG analysis identified classic *MAPK/WNT/ERBB* pathways are mostly altered in PM, suggesting that cfDNA of PM harbored similar molecular features of CRC. Oncogenic drivers of CRC focused in this study are typically truncal mutations. The high specificity of peritoneal cfDNA shows its utility to guide treatment independent of tissue sampling [19]. cfDNA mutations on driver genes can serve as predictors of disease burden and predict prognosis of PM. In RAS mutant tumor, RAS plasma mirror clinical and radiological response to chemo regimes and antiangiogenic drugs [11]. The resistance mechanisms of ERBB amplification can also guide development of novel targeted therapies, as drug resistance are common phenomenon in late-stage tumor, which highlight the importance to identify potential actionable resistance mechanisms in PM [21].

Despite the clinical values of our results, several limitations are still existing in this study. Firstly, this is a prospective diagnostic study with small sample size from one single institution. Our promising findings require to be verified in large cohorts of PM from multi centers. Secondly, although cfDNA experimental personals are blinded while clinicians know the diagnosis, selection of patients can still introduce bias. It is perfect if clinicians are blinded and they found peritoneal cfDNA without knowing intraoperative findings. In addition, patient population is heterogeneous as some of them received preoperative treatment which may impact some results. Thirdly, matched plasma cfDNA from WBC was used to match tumor tissue DNA to filter out somatic mutations.

In conclusion, our data show that peritoneal cfDNA detected using ultradeep NGS is a potential sensitive and reliable biomarker for detection of PM in CRC patients. It offers a minimally invasive tool for assessment of driver gene mutations to guide selection of candidate patients for targeted therapies. Peritoneal cfDNA can predict the prognosis of PM and serve as a potential surrogate or adjuvant biomarker in active postoperative surveillance of peritoneal dissemination in high-risk CRC patients instead of laparoscopic explore.

## Methods

### Patients and designs

This was a prospectively, open-label, blind, and diagnostic study. Patients with CRC were recruited from the Sixth Affiliated Hospital of Sun Yat-sen University, China. All enrolled patients underwent surgical resection of primary tumor or conduct CRS procedure. CRC and PM were diagnosed by pathology. Peritoneal lavage fluids were collected at the start of surgery. After surgery, CRC patients with PM were enrolled in the experimental PM arm, while CRC patients without PM were included in the controlled non-PM arm. cfDNA experimental personnel and statisticians were blinded to the diagnosis of PM, while clinicians know the diagnosis. For stage IV cases, metastatic lesions such as liver or lung sites were preoperatively radically resected, or treated by radiofrequency ablation. Extensive PM with PCI > 20 was defined as late PM, which is not appropriate for CRS plus HIPEC according to previous studies [5, 6].

In the training cohort, the diagnostic power of peritoneal cfDNA by ultradeep NGS was investigated. A validation cohort was enrolled to verify the results. All enrolled patients were consented for the collection of peritoneal lavages, tumor tissue, blood and medical records under the protocols approved by local ethical institutional review board (IRB), which was in accordance with the Declaration of Helsinki and later amendments. Written informed consents were obtained from all enrolled patients. All authors reviewed and approved the final manuscript. The follow-up was conducted until at least one year after surgery by professional personnel.

### Inclusion and exclusion criteria

Key inclusion criteria were listed as follows: consecutive patients with diagnosis of CRC and peritoneal metastasis by pathology, preoperative imaging for tumor staging, and Eastern Cooperative Oncology Group (ECOG) performance status of 0–2. Exclusion criteria included insufficient tumor tissue or peritoneal lavages, failure of NGS assays, missing data or follow-up and ineligible for cfDNA analysis.

The primary objective was to evaluate the sensitivity and specificity of peritoneal cfDNA in diagnosis of PM. The secondary objectives included positive/negative predictive values, accuracy, genomic mutation profiles in peritoneal cfDNA and matched tumor tissues, the underlying pathways and prognostic roles. The sample size was set as 1:1 ratio with each 20 cases in both arms.

### Sample collection, DNA isolation, and library preparation

Peritoneal lavage was collected after entering abdominal cavity during primary cancer surgery by laparotomy or laparoscopy in all enrolled patients. 300 ml of normal saline was infused into abdominal cavity through a small opening in the abdominal wall or laparoscopic trocar. About 100 ml of peritoneal lavage was aspirated for cfDNA sequencing. Then, resection of primary tumor was performed by surgeons. Peripheral blood was collected and saved in anticoagulant blood collection tube (EDTA tubes) in routine temperature. Tumor tissue specimens were obtained from resected tumor tissues. The percent of cancer cells by HE staining was ≥ 20%. If not, tumor mutational burden (TMB), CNV and microsatellite instability (MSI) tests may be affected.

DNA isolation and targeted sequencing were performed in Burning Rock Biotech (Shanghai, China), according to optimized protocols described previously [13, 22]. Briefly, cfDNA or tissue DNA was extracted from flash-frozen tissues using QIAamp DNA tissue kit according to manufacturer’s protocols (Qiagen). At least 30 ng DNA were extracted. cfDNA was recovered from 100 ml of peritoneal lavage fluids using the QIAamp Circulating Nucleic Acid kit (Qiagen). Briefly, a minimum of 50 ng gDNA was required for NGS library construction. DNA was sheared using Covaris M220 (Covaris, MA, USA). Fragments between 200 and 400 bp from the sheared DNA and DNA were purified by Agencourt AMPure XP Kit followed by end repair and a-tailing (Beckman Coulter). The DNA library preparation was constructed and the fragment of DNA was from 280 to 500 bp. The quality control of DNA and sequencing data were performed for each sample. Only qualified samples were stored for subsequent assays.

### Ultradeep NGS sequencing

The DNA samples from tumor tissue were ligated with regular adapters. Target capture of tissue DNA was performed using a commercial panel which consists of 520 genes (OncoScreen Plus), spanning 1.64 Mb of the human genome. The detailed genes were listed in Additional file 1: Table S4. Targeted gene regions included exons, introns, and promotor regions and MSI test. The gene panel consisted of commonly mutated genes in CRC which were correlated with tumor development and metastasis. These genes are chosen from one commercial cancer panel of Burning Rock Biotech (China). The quality and the size of fragments were evaluated by a highly sensitive DNA kit using Bioanalyzer 2100 (Agilent Technologies). Indexed samples were sequenced on Nextseq 500 (Illumina) with paired-end reads and average sequencing depth of 1,000 × for tissue samples.

The cfDNA from peritoneal lavage were ligated with UMI-containing adapters. Oligonucleotides corresponding to the UMI adapters were designed and patented by Burning Rock Biotech (China). The oligonucleotides were commercially synthesized, annealed and purified following standard protocols in previous study [23]. After purification using Agencourt AMPure XP Kit (Beckman Coulter, USA), the adapter-ligated DNA underwent hybridization with capture probes baits, hybrid selection with magnetic beads and PCR amplification. The quality and the size of the fragments were assessed by high sensitivity DNA kit using Bioanalyzer 2100 (Agilent Technologies, USA). Indexed samples were sequenced on Nextseq500 (Illumina, Inc., USA) with paired-end reads and average sequencing depth of 35,000X for UMI-tagged cfDNA samples. The workflow details of cfDNA assay were listed in previous study [19].

### Analysis of NGS sequencing data

Sequencing data were mapped to the reference of human genome (hg19) using Burrows-Wheeler Aligner version 0.7.10 [24]. Local alignment optimization, duplication marking and variant calling were performed using Genome Analysis Tool Kit version 3.2 [25], and VarScan version 2.4.3 [26]. Tissue samples were compared to white blood cell to filter out germline variants or clonal hematopoiesis [20]. Variants were filtered using the VarScan fpfilter pipeline. Loci with depth < 100 were filtered out. Base calling in plasma and tissue samples required at least eight supporting reads for SNV, two, and five supporting reads for insertion-deletion variations (Indels), respectively. Variants with population frequency > 0.1% in the ExAC, 1000 Genomes, dbSNP or ESP6500SI-V2 databases were grouped as SNP and were excluded. Remaining variants were annotated with ANNOVAR (2016–02-01 release) [27] and SnpEff version 3.6 [28]. Analysis of structural variations was performed using Factera version 1.4.3 [29].

### Gene enrichment analysis

Gene enrichment analysis was performed using the GSEA software program [30] and Kyoto Encyclopedia of Genes and Genomes (KEGG) gene set annotations downloaded from MSigDB (http://www.broadinstitute.org/gsea/msigdb/index.jsp). The parameters we applied were hypergeometric algorithm and Benjamini-Hochberg correction for multiple testing.

### Definitions

The cutoff value of positivity threshold in peritoneal cfDNA was calculated according to MaxAF by ROC analyses, which allowed the best discrimination between PM and non-PM [9]. The MaxAF value was defined as the maximum value of all variants in the sample, excluding fusion, CNV and LGR variants. The distribution of MaxAF value in the blood of CRC patients from the company bio-database was used to tune the detection cutoff value for cfDNA [10] in a large scale of > 1000 people stored in the bio-database previously of Burning Rock Biotech (China). The MaxAF value of cfDNA was defined as the maximum value of mutant allele fractions for all mutations derived from the patients. RFS was defined as the interval time from radical surgery to tumor recurrence or last follow-up.

CNVs were analyzed based on the depth of coverage data of capture intervals. Coverage data were corrected against sequencing bias from GC content and probe design. The average coverage of all captured regions was used to normalize the coverage of different samples to comparable scales. Copy number was calculated based on the ratio between the depth of coverage in tumor samples and average coverage of an adequate number (*n* > 50) of samples without CNVs as references per capture interval. CNV is defined as the coverage of gene region was significant different from the reference control. The limit level of CNVs detection is 1.75 for copy number deletion and 2.75 for copy number amplifications.

### Statistical analysis

All analysis was conducted using the SPSS software (v.15.0; SPSS Inc. Chicago, IL) in this study. Student's *t* test, Fisher’s exact test, *x*^*2*^ test, Mann–Whitney *U* test, one-way ANOVA, and Pearson correlation were used for comparisons of different variables. The unweighted Cohen’s kappa coefficient was assessed for the concordance between peritoneal cfDNA and tissue DNA. Cox proportional hazards regression was conducted for univariate and multivariate analysis of risk factors of survival. Kaplan-Meier method with log-rank test and the Cox exp (beta) were used to estimate hazard ratios of RFS between groups. ROC curves and AUC were performed for sensitivity and specificity. *P* value < 0.05 (two-sided) was considered to be statistically significant.

## Supplementary Information

**Additional file 1**. **Table S1 **The comparisons of clinicopathological characteristics of PM and non-PM in the study. **Table S2 **The univariant and multivariant analyses of risk factors of RFS in PM. **Table S3 **The details of KEGG enrichment analysis were listed. **Table S4 **The details of the 520 gene panel were listed.

**Additional file 2.** **Figure S1 **The sequencing depth and DNA insert sizes fulfilled the quality of control in both FLD (over 30,0000X) and TIS (over 1000X) (A). The concentration of DNA was lower in non-PM than PM (*P*<0.005) (D). The mean insert size was non-significant higher in non-PM than PM (*P*=0.055) (E). **Figure S2** Genetic mutational profiling of NGS in TIS of CRC were compared between PM and non-PM in the training cohort. The frequencies of driver mutant KRAS, BRAF, TP53, APC, PIK3CA, and SMAD4 were 52%, 10%, 80%, 62%, 18% and 18%, respectively. **Figure S3** The mutational profiling of ultra-deep NGS in FLD were compared between PM and non-PM in the training cohort. The frequencies of driver mutant KRAS, BRAF, TP53, APC, PIK3CA, and SMAD4 were 32%, 10%, 45%, 38%, 5% and 22%, respectively. The overall mutation frequencies were lower than TIS due to very low mutations detected in FLD of non-PM patients. **Figure S4** The shared SNV/Indel variants of 58.9% were shown both in FLD and TIS with sensitivity of 72% in PM patients. **Figure S5** The lollipop plots of driver mutations and chr20q mutations in the tumor tissues of PM and non-PM. (A)Suppressor TP53 mutations were detected in 76.5% (13/17) in TIS of PM, comparing to 82.6% (19/23) in non-PM. (B)47.1% (8/17) of APC mutations were identified in TIS of PM, comparing to 73.9% (17/23) in the non-PM. (C)Amplification in Chr20q (MSS-A) were observed in 81.8% (18/22) of non-PM, while 100% (17/17) of PM were MSS-N (P=0.118). **Figure S6** The overall MaxAF values could not be affected by single driver mutation in FLD of PM. (A-F) The average MaxAF is not changed (*P*>0.05) in wild and mutant driver genes in the FLD of PM, including KRAS, BRAF, TP53, APC, PIK3CA, and SMAD4. (G-K) In the FLD of control, higher MaxAF were observed in mutant TP53 (*P*=0.02), mutant APC (*P*<0.01) and SMAD4 (*P*=0.05) than wild types due to overall low mutant frequencies in non-PM. **Figure S7** Altered cellular pathways by KEGG enrichment analysis in PM and non-PM. The top signaling of mutational genes were comparable in PM and non-PM, including MAPK, WNT, and ERBB signaling. **Figure S8** The cutoff value of FLD MaxAF were identified at best positivity threshold of 6.29% by ROC analyses and distribution of MaxAF in the blood of large scale population, representing. (A-B) The majority of PM were over this cutoff value with positivity, while control obtained negativity below this cutoff value of 6.29%. (C-D) The density of blood MaxAF was stable above this cutoff MaxAF in more than 1000 cases of CRC patients in the bio-database of Burning Rock company. In addition, the distribution of positive MaxAF 6.29% located in the upper quartile.

## Data Availability

The data can be available if requested according to the journal instructions.
