# Supplementary material for: Peritoneal cell-free DNA as a sensitive biomarker for detection of peritoneal metastasis in colorectal cancer: a prospective diagnostic study
Source: Clin Epigenetics. 2023 Apr 18;15:65. doi: 10.1186/s13148-023-01479-9 (PMC10114319; doi:10.1186/s13148-023-01479-9)
Supplement: Supplementary file 1 — Additional file 1. Table S1 The comparisons of clinicopathological characteristics of PM and non-PM in the study. Table S2 The univariant and multivariant analyses of risk factors of RFS in PM. Table S3 The details of KEGG enrichment analysis were listed. Table S4 The details of the 520 gene panel were listed. [file 13148_2023_1479_MOESM1_ESM.docx]

**Table S1** **The comparisons of clinicopathological characteristics of PM and non-PM in the study.**

|  | | | Overall  (n = 51) | | CRC patients with PM  (n = 23) | | CRC patients without PM  (n = 28) | | *P* values | |
| --- | --- | --- | --- | --- | --- | --- | --- | --- | --- | --- |
| Age | | |  | |  | |  | | 0.074 | |
| Mean(SD) | | 53.35 (10.90) | | 50.35 (12.44) | | 55.82 (8.94) |  |  |  |  |
| Location | |  | |  | |  | 0.015 | |  |  |
| Left | | 35 (68.6) | | 12 (52.2) | | 23 (82.1) |  |  |  |  |
| Right | | 15 (29.4) | | 11 (47.8) | | 4 (14.3) |  |  |  |  |
| NA | | 1 (2.0) | | 0 (0.0) | | 1 (3.6) |  |  |  |  |
| Gender | |  | |  | |  | 0.042 | |  |  |
| female | | 31 (60.8) | | 18 (78.3) | | 13 (46.4) |  |  |  |  |
| male | | 20 (39.2) | | 5 (21.7) | | 15 (53.6) |  |  |  |  |
| Histological type | |  | |  | |  | 0.311 | |  |  |
| Well-differentiated adenocarcinoma | | 39 (76.5) | | 16 (69.6) | | 23 (82.1) |  |  |  |  |
| Signet ring cell carcinoma&Mucinous adenocarcinoma&poor differentiation | | 10 (19.6) | | 6 (26.1) | | 4 (14.3) |  |  |  |  |
| Resection of primary tumour | |  | |  | |  | <0.001 | |  |  |
| Yes | | 32 (62.7) | | 6 (26.1) | | 26 (92.9) |  |  |  |  |
| No | | 19 (37.3) | | 17 (73.9) | | 2 (7.1) |  |  |  |  |
| TNM_T | |  | |  | |  | | | 0.303 | |
| Tis-T2 | | 6 (11.8) | | 0 (0.0) | | 6 (21.4) | | |  |  |
| T3-4 | | 30 (58.8) | | 9 (39.1) | | 21 (75.0) | | |  |  |
| NA | | 15 (29.4) | | 14 (60.9) | | 1 (3.6) | | |  |  |
| TNM_N | |  | |  | |  | | | 0.292 | |
| N0 | | 20 (39.2) | | 3 (13.0) | | 17 (60.7) | | |  |  |
| N1 | | 9 (17.6) | | 3 (13.0) | | 6 (21.4) | | |  |  |
| N2 | | 7 (13.7) | | 3 (13.0) | | 4 (14.3) | | |  |  |
| NA | | 15 (29.4) | | 14 (60.9) | | 1 (3.6) | | |  |  |
| pTNM_M | |  | |  | |  | | | <0.001 | |
| M0 | | 21 (41.2) | | 0 (0.0) | | 21 (75.0) | | |  |  |
| M1 | | 29 (56.9) | | 23 (100.0) | | 6 (21.4) | | |  |  |
| NA | | 1 (2.0) | | 0 (0.0) | | 1 (3.6) | | |  |  |
| PCI scores | |  | |  | |  | | | <0.001 | |
| Mean(SD) | | 9.43 (13.29) | | 20.91 (12.25) | | 0.00 (0.00) | | |  | |
| CA125 | |  | |  | |  | | | 0.004 | |
| Mean(SD) | | 57.73 (107.93) | | 104.80 (146.62) | | 19.06 (24.82) | | |  |  |
| CA199 | |  | |  | |  | | | 0.103 | |
| Mean(SD) | | 344.24 (1518.37) | | 727.00 (2226.26) | | 29.83 (60.29) | | |  |  |
| CEA | |  | |  | |  | | | 0.054 | |
| Mean(SD) | | 74.66 (223.28) | | 141.36 (315.09) | | 15.97 (31.88) | | |  |  |

**Table S2 The univariant and multivariant analyses of risk factors of RFS in CRC with PM.**

| **Variable** | **Univariant analysis** | | **Multivariant analysis** | |
| --- | --- | --- | --- | --- |
|  | **HR** | **P** | **HR** | **P** |
| Gender:Male versus Female | 5.50 | 0.087 | 1.94 | 0.62 |
| Location:Right versus Left | 1.49 | 0.720 | 14.77 | 0.177 |
| TNM_T:T3-T4 versus T0-T2 | 2.52E+08 | 0.275 |  |  |
| TNM_N:N1-N2 versus N0 | 1.19 | 0.849 | 4.92 | 0.352 |
| CEA>5:Yes versus No | 4.77 | 0.126 |  |  |
| **MaxAF:Positive versus Negative** | 7.1 | 0.013 | 22.33 | 0.044 |
| Chr20q:Amp versus Normal | 1.49 | 0.720 | 0.32 | 0.577 |
| TP53:Mut versus Normal | 5.81 | 0.031 | 12.07 | 0.066 |
| APC:Mut versus Normal | 4.07 | 0.096 | 1.85 | 0.559 |
| KRAS:Mut versus Normal | 1.53 | 0.702 |  |  |

**Table S3 The details of KEGG enrichment analysis were listed.**

**Table S4** **The details of the 520 gene panel were listed.**
