# Supplementary material for: Peritoneal cell-free DNA as a sensitive biomarker for detection of peritoneal metastasis in colorectal cancer: a prospective diagnostic study
Source: Clin Epigenetics. 2023 Apr 18;15:65. doi: 10.1186/s13148-023-01479-9 (PMC10114319; doi:10.1186/s13148-023-01479-9)

**Figure S1** The sequencing depth and DNA insert sizes fulfilled the quality of control in both FLD (over 30,0000X) and TIS (over 1000X) (A-C).

The concentration of DNA were lower in non-PM than PM(D). The mean insert size was non-significant higher in non-PM than PM (E).


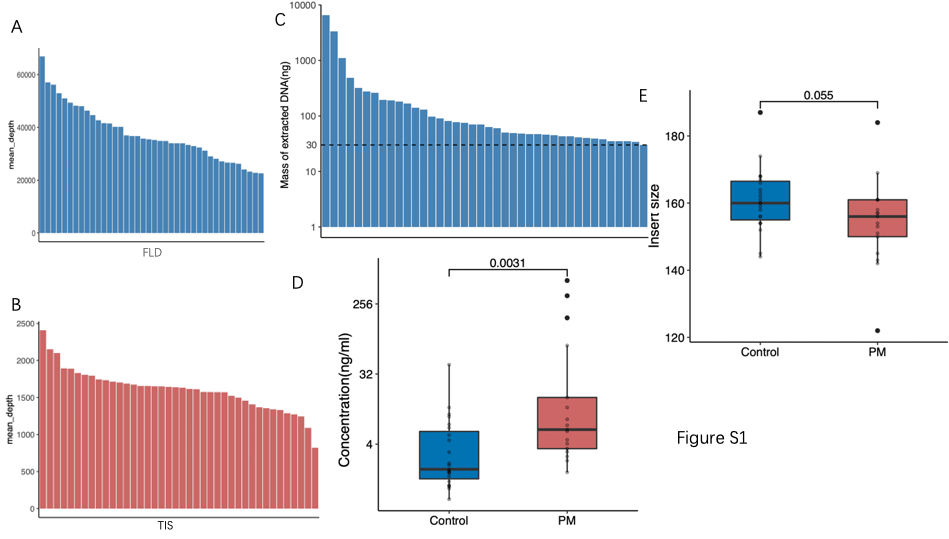


**Figure S2** Genetic mutational profiling of NGS in TIS of CRC were compared between PM and non-PM in the training cohort.

The frequencies of driver mutant KRAS, BRAF, TP53, APC, PIK3CA, and SMAD4 were 52%, 10%, 80%, 62%, 18% and 18%, respectively.


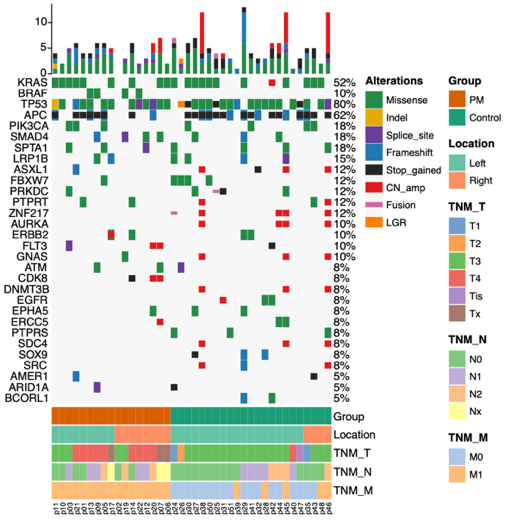


**Figure S3** The mutational profiling of ultra-deep NGS in FLD were compared between PM and non-PM in the training cohort. The frequencies of driver mutant KRAS, BRAF, TP53, APC, PIK3CA, and SMAD4 were 32%, 10%, 45%, 38%, 5% and 22%, respectively. The overall mutation frequencies were lower than TIS due to very low mutations detected in FLD of non-PM patients.


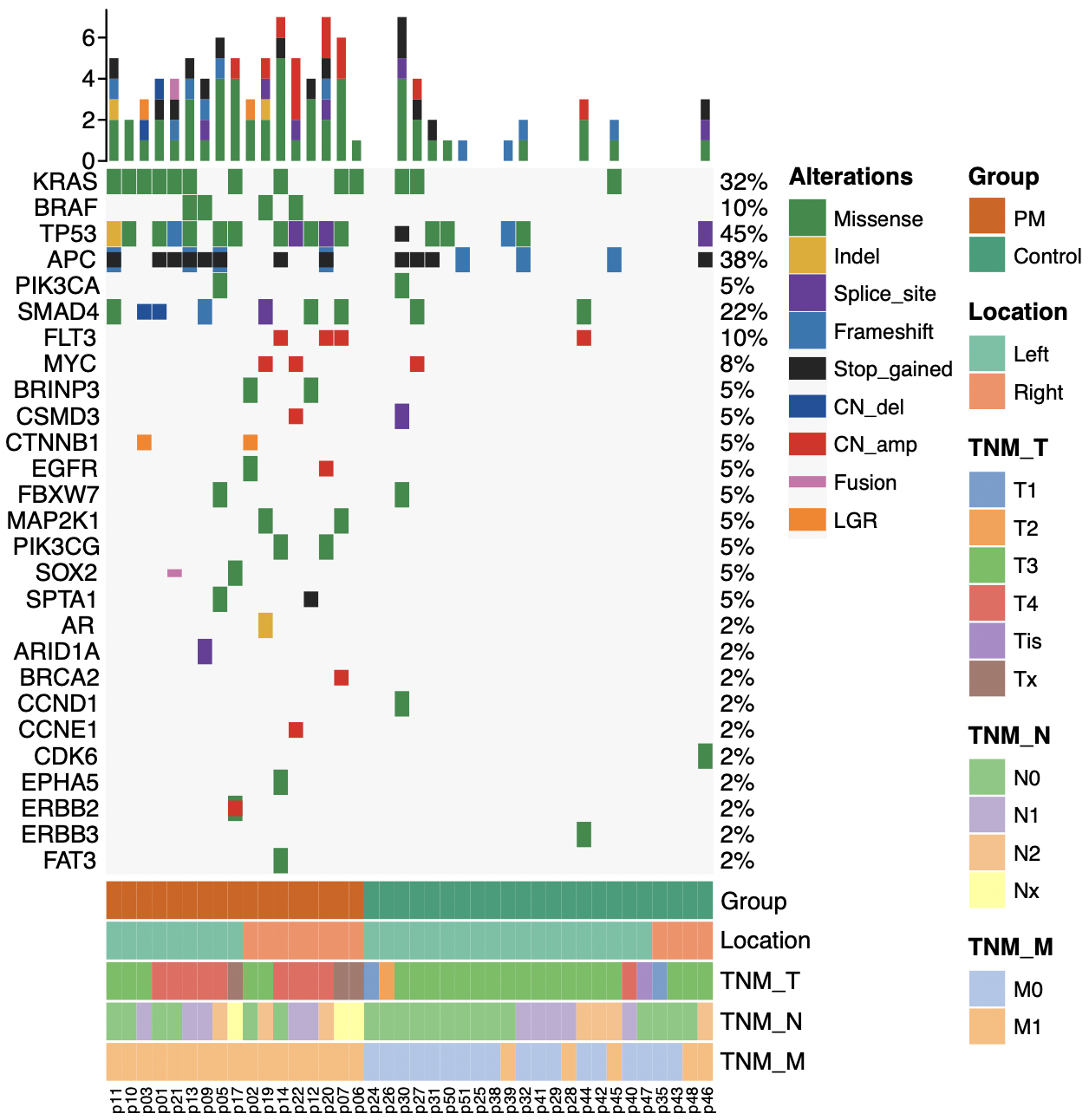


**Figure S4** The shared SNV/Indel variants of 58.9% were shown both in FLD and TIS with sensitivity of 72% in PM patients.


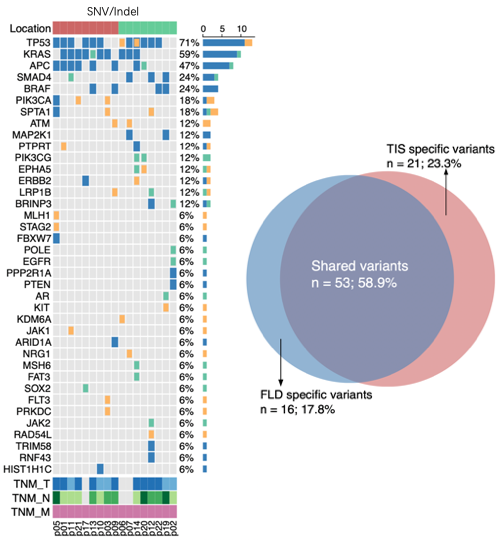


**Figure S5** The lollipop plots of driver mutations and chr20q mutations in the tumor tissues of PM and non-PM. (A) Suppressor TP53 mutations were detected in 76.5% (13/17) in TIS of PM, comparing to 82.6% (19/23) in non-PM. (B) 47.1% (8/17) of APC mutations were identified in TIS of PM, comparing to 73.9% (17/23) in the non-PM. (C) Amplification in Chr20q (MSS-A) were observed in 81.8% (18/22) of non-PM, while 100% (17/17) of PM were MSS-N (*P*=0.118).


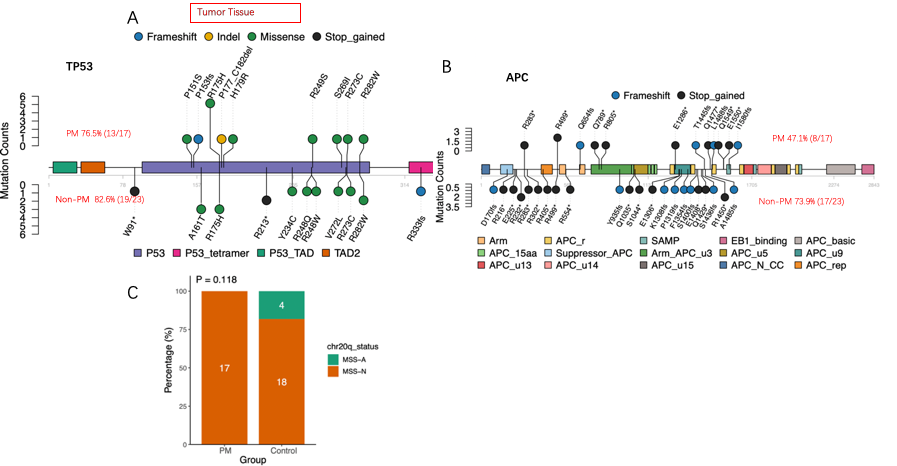


**Figure S6** The overall MaxAF values could not be affected by single driver mutation in FLD of PM. (A-F) The average MaxAF is not changed (P>0.05) in wild and mutant driver genes in the FLD of PM, including KRAS, BRAF, TP53, APC, PIK3CA, and SMAD4. (G-K) In the FLD of control, higher MaxAF were observed in mutant TP53 (*P*=0.02), mutant APC (*P*<0.01) and SMAD4 (*P*=0.05) than wild types due to overall low mutant frequencies in non-PM.


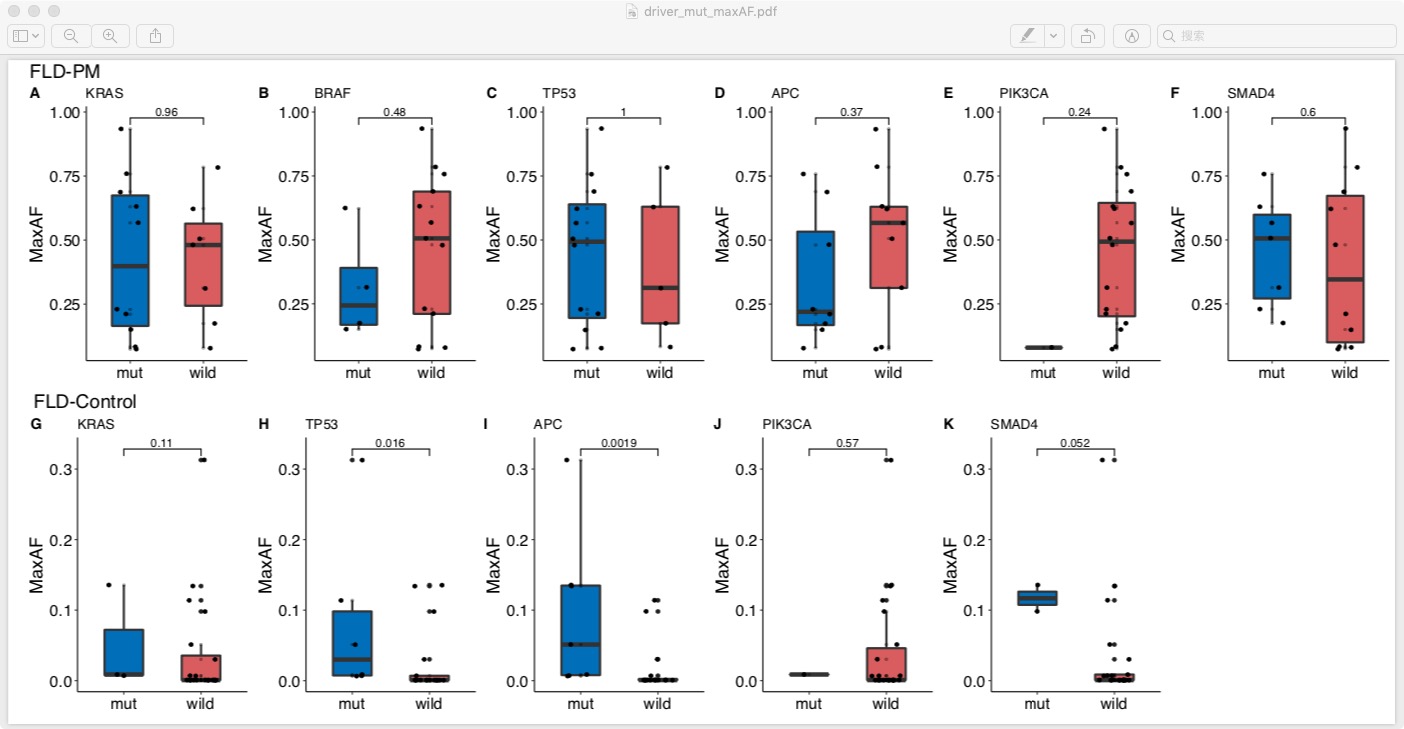


**Figure S7** Altered cellular pathways by KEGG enrichment analysis in PM and non-PM. The top signaling of mutational genes were comparable in PM and non-PM, including MAPK, WNT, and ERBB signaling.


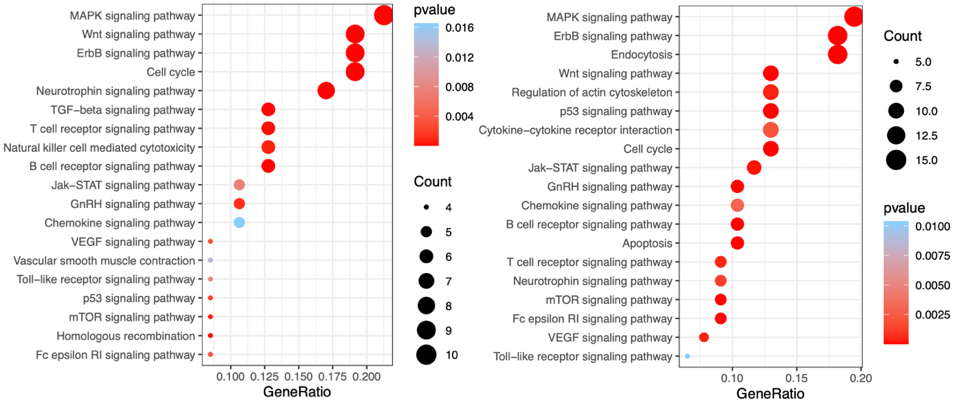


**Figure S8** The cutoff value of FLD MaxAF were identified at best positivity threshold of 6.29% by ROC analyses and distribution of MaxAF in the blood of large scale population, representing. (A-B) The majority of PM were over this cutoff value with positivity, while control obtained negativity below this cutoff value of 6.29%. (C-D) The density of blood MaxAF was stable above this cutoff MaxAF in more than 1000 cases of CRC patients in the bio-database of Burning Rock company. In addition, the distribution of positive MaxAF 6.29% located in the upper quartile.


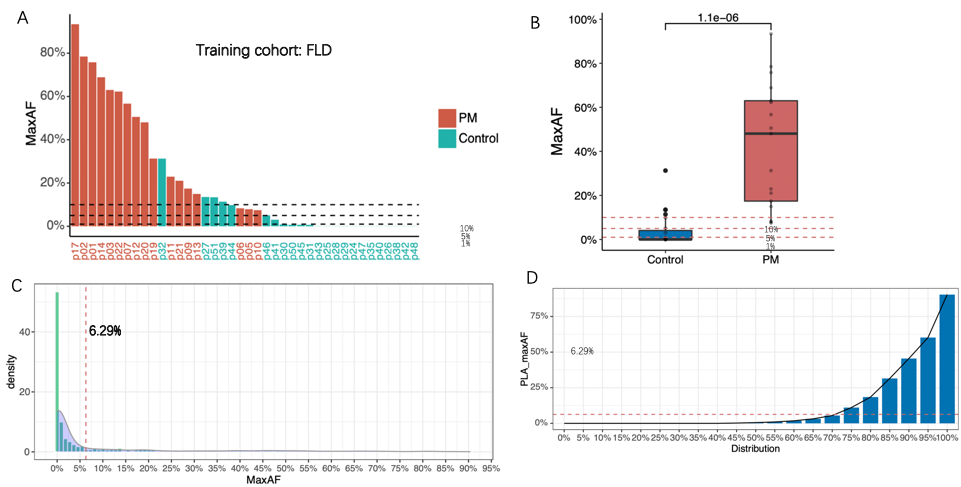


**Figure S9** Positive cfDNA in FLD can predict poor prognosis in stage III of CRC patients. (A) Positive cfDNA in FLD was associated with tumor relapse in stage III of CRC (*P*=0.043); (B) The follow up of tumor relapse in non-PM patients.


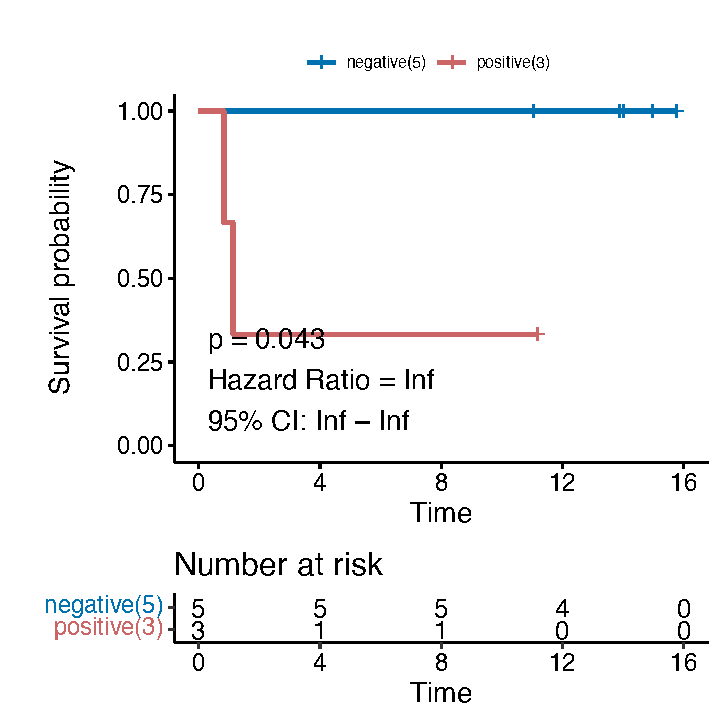

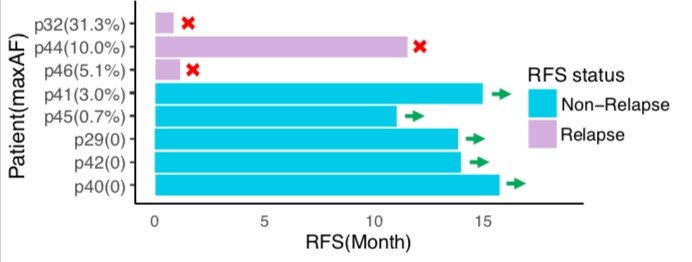

Supplement: Supplementary file 2 — Additional file 2. Figure S1 The sequencing depth and DNA insert sizes fulfilled the quality of control in both FLD (over 30,0000X) and TIS (over 1000X) (A). The concentration of DNA was lower in non-PM than PM (P<0.005) (D). The mean insert size was non-significant higher in non-PM than PM (P=0.055) (E). Figure S2 Genetic mutational profiling of NGS in TIS of CRC were compared between PM and non-PM in the training cohort. The frequencies of driver mutant KRAS, BRAF, TP53, APC, PIK3CA, and SMAD4 were 52%, 10%, 80%, 62%, 18% and 18%, respectively. Figure S3 The mutational profiling of ultra-deep NGS in FLD were compared between PM and non-PM in the training cohort. The frequencies of driver mutant KRAS, BRAF, TP53, APC, PIK3CA, and SMAD4 were 32%, 10%, 45%, 38%, 5% and 22%, respectively. The overall mutation frequencies were lower than TIS due to very low mutations detected in FLD of non-PM patients. Figure S4 The shared SNV/Indel variants of 58.9% were shown both in FLD and TIS with sensitivity of 72% in PM patients. Figure S5 The lollipop plots of driver mutations and chr20q mutations in the tumor tissues of PM and non-PM. (A)Suppressor TP53 mutations were detected in 76.5% (13/17) in TIS of PM, comparing to 82.6% (19/23) in non-PM. (B)47.1% (8/17) of APC mutations were identified in TIS of PM, comparing to 73.9% (17/23) in the non-PM. (C)Amplification in Chr20q (MSS-A) were observed in 81.8% (18/22) of non-PM, while 100% (17/17) of PM were MSS-N (P=0.118). Figure S6 The overall MaxAF values could not be affected by single driver mutation in FLD of PM. (A-F) The average MaxAF is not changed (P>0.05) in wild and mutant driver genes in the FLD of PM, including KRAS, BRAF, TP53, APC, PIK3CA, and SMAD4. (G-K) In the FLD of control, higher MaxAF were observed in mutant TP53 (P=0.02), mutant APC (P<0.01) and SMAD4 (P=0.05) than wild types due to overall low mutant frequencies in non-PM. Figure S7 Altered cellular pathways by KEGG enrichment analysis in PM and non-PM. The [file 13148_2023_1479_MOESM2_ESM.docx]
